# Association of *HNF1A* gene variants and haplotypes with metabolic syndrome: a case–control study in the Tunisian population and a meta-analysis

**DOI:** 10.1186/s13098-022-00794-0

**Published:** 2022-02-02

**Authors:** Hamza Dallali, Meriem Hechmi, Imane Morjane, Sahar Elouej, Haifa Jmel, Yosra Ben Halima, Abdelmajid Abid, Afef Bahlous, Abdelhamid Barakat, Henda Jamoussi, Sonia Abdelhak, Rym Kefi

**Affiliations:** 1 Laboratory of Biomedical Genomics and Oncogenetics, Institut Pasteur in Tunis, BP 74, 13 Place Pasteur, Belvedere, 1002 Tunis, Tunisia; 2 University of Carthage, National Institute of Applied Science and Technology, Tunis, Tunisia; 3 Human Molecular Genetics Laboratory, Institut Pasteur du Maroc, Place Louis Pasteur, Casablanca, Morocco; 4 National Institute of Nutrition and Food Technology, 11 rue Jebel Lakhdar, Bab Saadoun, 1007 Tunis, Tunisia; 5 Central Laboratory of Medical Biology, Institut Pasteur in Tunis, 13 Place Pasteur, BP 74, 1002 Tunis, Tunisia; 6 University of Tunis El Manar, 2092 El Manar I Tunis, Tunisia

**Keywords:** Hepatocyte Nuclear Factor-1-Alpha gene, Metabolic disorders, Lipids, SNP, Haplotype, North Africa

## Abstract

**Background:**

Variants in the Hepatocyte Nuclear Factor 1 Alpha gene (*HNF1A*) are associated with lipoproteins levels and type 2 diabetes. In this study, we aimed to assess the association of *HNF1A* gene and haplotypes with the metabolic syndrome (MetS) and its components through an association study in the Tunisian population as well as by a meta-analysis.

**Methods:**

A total of 594 Tunisian individuals were genotyped for three variants (rs1169288, rs2464196 and rs735396) located in *HNF1A* gene using KASPar technology. Statistical analyses were performed with R software. The association was furthermore evaluated through a meta-analysis of our results with those obtained in a Moroccan population.

**Results:**

Our results showed no association between *HNF1A* variants and MetS in the Tunisian population. However, a significant association was observed between the variant rs735396 and a higher waist circumference. The stratified analysis according to the sex highlighted a significant association between the variant rs1169288 and high cholesterol levels only in women. Similarly, Haplotype analysis showed an association between the *HNF1A* minor haplotype and high total cholesterol mainly in women. Finally, our meta-analysis showed no association between *HNF1A* variants and MetS.

**Conclusions:**

Our findings exclude the involvement of the three *HNF1A* variants rs1169288, rs2464196 and rs735396 in the susceptibility to MetS in our studied Tunisian population but emphasize the role of these variants in the cholesterol homeostasis with sex-specific differences, which may serve to rise clinical consideration to early statin therapy in women carrying these genetic variants.

**Supplementary Information:**

The online version contains supplementary material available at 10.1186/s13098-022-00794-0.

## Introduction

Metabolic syndrome (MetS) is characterized by the clustering of risk factors for heart disease and type 2 diabetes (T2D). These risk factors include central obesity, hyperglycemia, hypertension, and altered lipid profile [1, 2].

The prevalence of MetS has reached levels comprised between 20 and 40% across populations in the last two decades [3]. In Tunisia, the prevalence is 30% of the population according to the last epidemiological study carried out in 2013 [4]. Consequently, MetS represents a heavy burden on the human public health system both in developing and developed countries. MetS is a complex multifactorial syndrome. Environmental factors such as low physical activity and hypercaloric diet are potential determinants of MetS [4, 5]. The genetic component is also crucial since MetS incidence is significantly higher in individuals with family history [6, 7].

Genome wide association studies (GWAS) have identified loci associated with MetS as an overall entity, or with some of its phenotypic traits or its complications [8, 9]. In 2009, Kathiresan et al. conducted genome wide association screens in 19,840 individuals and replication in up to 20,623 individuals in order to investigate the polygenic basis of the dyslipidemia traits. They highlighted single nucleotide polymorphisms (SNPs) in 30 loci associated with lipoproteins and triglycerides levels, of which 11 loci reaching genome wide significance (p < 5.10^–8^) for the first time. These 11 loci include genes such as Hepatocyte Nuclear Factor 1 Alpha (*HNF1A*) [10].

*HNF1A* (MIM*142410) is located on chromosome 12q24.2 and counts 10 exons. The protein encoded by this gene is a transcription factor required for the expression of at least 222 liver-specific genes that are essential in the carbohydrate synthesis and storage as well as in lipid metabolism (synthesis of cholesterol and lipoproteins) [11]. Defects in *HNF1A* gene are known to cause maturity onset diabetes of the young type 3 (MIM#600496). This form of diabetes has an autosomal dominant inheritance, and it is characterized by severe hyperglycemia caused by beta cells insulin secretion deficit, and an age of onset generally younger than 25 years [12].

Some studies were carried out to test the association of *HNF1A* gene variants with metabolic disorders. In 2011, Avery et al. conducted a GWAS including 19,486 European American and 6287 African American and they detected the association of a *HNF1A* variant with a phenotypic cluster consisting of atherogenic dyslipidemia, vascular inflammation and prothombotic state [9]. Other studies have detected associations of *HNF1A* variants with some MetS components including altered lipid profile and its complications in different populations [13–22].

Among these reported variants, we selected for our study three SNPs: rs1169288 located in the exon 1 also known as I27L, rs2464196 located in the exon 7 also called S487N, and rs735396 located in the intron 9.

The main goals of the present study are: (1) to investigate the association of *HNF1A* gene variants (rs1169288, rs2464196 and rs735396) and haplotypes with the susceptibility to MetS and its components in the Tunisian population, (2) to perform a meta-analysis of the association between *HNF1A* variants and MetS. Our study is the third to test association of *HNF1A* variants with MetS, and it includes the first meta-analysis to date of this association. These variants were previously associated with MetS, altered lipoproteins levels and type 2 diabetes [15, 16, 18, 20, 23, 24].

## Materials and methods

### Study subjects

This study is conducted in the frame of MEDIGENE project, that was approved by the Ethical committee of Institut Pasteur in Tunis (Reference IPT/LR11-05/Etude 04/2013) [25]. A total of 594 participants (299 controls and 295 MetS patients), aged between 35 and 75 years, were recruited and clinically characterized as previously described [26, 27].

### Genetic analysis

Genomic DNA was isolated from the whole blood. Genotyping of SNPs was performed by KASPar® technology (*KBioscience, UK*) using the LightCycler 480® system (*Roche Diagnostics, Switzerland*) [28]. A random of 10% sample set was re-tested with the same method to confirm genotype accuracy.

### Statistical analysis

The power analysis of the case–control study was performed using PS: Power and Sample Size Calculations software version (3.1.2) [29].

The measured clinical features were expressed as means ± standard deviations, and differences between groups were assessed with Student Test.

The Hardy Weinberg equilibrium (HWE) was checked for each of the genotyped SNPs. Allelic and genotypic frequencies for the three SNPs were calculated in the studied population. The associations of the genotyped SNPs with MetS were estimated using multivariate logistic regression model after adjustment for age, sex and body mass index (BMI). Linear regression analysis was performed to identify the associations between the variants and the measured quantitative traits. The association tests were performed under three different genetic models: codominant, dominant and recessive models of inheritance. Results were expressed as nominal p-values, odds ratios (OR), and 95% confidence intervals (CI). A p-value < 0.05 was considered statistically significant for statistical tests. The p-values were corrected with the Bonferroni correction by multiplying with the number of comparisons. Statistical analyses were performed using SNPassoc R package [30].

Linkage disequilibrium (LD) statistics was computed using r^2^ coefficient by Haploview software (version 4.2) [31]. Haplotype frequencies and associations with MetS were estimated using PLINK software (version 1.07) [32]. In addition, we explored the haplotype associations with the measured quantitative traits using a generalized linear model from the haplo.score function incorporated in the haplo.stats R package [33].

### Meta-analysis

Relevant studies, evaluating the associations between *HNF1A* polymorphisms and MetS, were identified by searching HuGe navigator (https://phgkb.cdc.gov/PHGKB/hNHome.action) and PubMed (https://www.ncbi.nlm.nih.gov/pubmed/) databases. We used different combinations of the following keywords: “*HNF1A*”, “metabolic syndrome”, “association” and “polymorphism”. The references of retrieved studies were inspected to identify any other relevant studies.

Association studies included in our meta-analysis had to meet the following criteria: (1) the MetS was defined according to the International Diabetes Federation criteria; (2) evaluation of association of *HNF1A* polymorphisms with MetS in at least two studies; (3) use of a case–control design; (4) the given information was sufficient to calculate the pooled odds ratio (OR), either a contingency table containing the number of controls and MetS cases with the different genotypes levels of the studied polymorphisms, or the raw genotyping data.

Statistical heterogeneity across studies was evaluated by using the chi-square based Q test and the I^2^ statistics [34]. If there is no heterogeneity across studies (Q test p-value > 0.1 or I^2^ < 50%), the fixed effects model of Mantel–Haenszel was conducted for the meta-analysis [35]. Otherwise, the random effects model of DerSimonian and Laird was used [36].

Statistical tests were performed using GWAMA software (version 2.2.2) [37]. The R library “rmeta” was used to draw the forest plots [38]. Publication bias was checked by using Begg’s test computed with the metaphor R package [39].

## Results

### Characteristics of the studied population

The biochemical and clinical data of the studied population are presented in Table 1.

**Table 1:** Clinical and biochemical characteristics of the studied Tunisian population

	Controls (n = 299)	Mets (n = 295)	p-value
Age (years)	52.56 ± 10.09	56.58 ± 8.56	< 0.001
WC (cm)	97.07 ± 11.87	106.50 ± 9.94	< 0.001
BMI (Kg/m^2^)	28.41 ± 4.83	31.53 ± 5.12	< 0.001
FPG (mmol/l)	6.13 ± 2.51	9.52 ± 4.28	< 0.001
TC (mmol/l)	5.08 ± 0.92	5.16 ± 1.01	0.28
HDL (mmol/l)	1.48 ± 0.41	1.13 ± 0.34	< 0.001
LDL (mmol/l)	3.12 ± 0.89	3.16 ± 1.34	0.049
TG (mmol/l)	1.29 ± 0.56	2.02 ± 0.93	< 0.001
DBP (mmHg)	7.74 ± 1.26	8.35 ± 1.40	< 0.001
SBP (mmHg)	13.20 ± 1.97	14.6 ± 2.1	< 0.001

*BMI* Body Mass Index, *DBP* diastolic blood pressure, *FPG* fasting plasma glucose, *HDL* high density lipoprotein cholesterol, *LDL* low density lipoprotein cholesterol, *SBP* systolic blood pressure, *TC* total cholesterol, *TG* triglycerides, *WC* waist circumference. Data are presented as mean ± standard deviation (SD)

The BMI, WC, TG, low density lipoprotein cholesterol (LDL), FPG, diastolic and systolic blood pressure were significantly higher in MetS patients compared to controls. An opposite result was observed only with HDL levels.

### Association with MetS

Based on the minor allele frequency of the genotyped SNPs in EXAC database (http://exac.broadinstitute.org/), the power analysis demonstrated that our study sample size (299 controls/295 cases) is sufficient to detect odds ratios ≃ 1.6, 1.603 and 1.592 for rs1169288, rs2464196 and rs735396 respectively with 80% of power at p < 0.05.

Genotypic success rates were 99.32% for rs1169288, 100% for rs2464196 and 99.5% for rs735396. The genotypic and allelic distribution of *HNF1A* variants (rs1169288, rs2464196 and rs735396) and the result of the association with MetS are shown in the Additional File 1. The three genotyped SNPs did not deviate from HWE, and their genotype distributions are not significantly different between MetS patients and controls in our Tunisian cohort.

In addition, we performed an association analysis after stratification of the studied population according to the sex. Our results showed no association with MetS neither for men nor for women (Additional file 2).

The whole sample was stratified into Northern and Southern groups in order to investigate the impact of the geographic origin on the genotype distribution of *HNF1A* variants. Association analyses of the three *HNF1A* variants with MetS performed for each group separately showed no significant associations under any genotypic model (Additional file 3). Furthermore, we did not find any significant difference in the distribution of *HNF1A* genotypes after stratification of the whole sample according to the sex and to the geographic origin (Additional file 4).

### Association with quantitative traits

The association analyses results of the *HNF1A* variants with different quantitative traits are shown in Table 2. We applied the recessive model since it has the lowest Akaike Information Criterion (AIC) value as shown in the additional file 1.

**Table 2:** Association of *HNF1A* variants with metabolic syndrome traits in the studied Tunisian population

	rs1169288				rs2464196				rs735396			
	AA + AC	CC	p-value	p-value*	GG + GA	AA	p-value	p-value*	TT + TC	CC	p-value	p-value*
WC (cm)	101.6	102.3	0.65	1	101.6	105.3	0.035^a^	0.105	101.5	104.3	0.013^a^	0.039^a^
BMI (kg/m^2^)	30.05	30.17	0.99	1	29.93	30.68	0.1	0.3	29.94	30.35	0.35	1
FPG (mmol/l)	7.81	8.68	0.1	0.3	7.8	8.36	0.47	1	7.9	7.88	0.85	1
SBP (mmHg)	14.01	13.81	0.48	1	13.95	14.11	0.62	1	13.98	13.95	0.72	1
DBP (mmHg)	8.08	8.08	0.99	1	8.07	8.08	0.61	1	8.11	7.97	0.27	0.81
TC (mmol/l)	5.09	5.31	0.056	0.168	5.08	5.30	0.056	0.168	5.11	5.13	0.83	1
HDL (mmol/l)	1.30	1.38	0.07	0.21	1.3	1.37	0.037^a^	0.111	1.29	1.36	0.08	0.24
LDL (mmol/l)	3.24	3.31	0.61	1	3.23	3.34	0.45	1	3.24	3.25	0.9	1
TG (mmol/l)	1.66	1.76	0.5	1	1.66	1.71	0.96	1	1.66	1.69	0.8	1

Data are presented as means. Linear regression, adjusted for age, sex and BMI, was used to assess genotype phenotype correlations under the recessive model of inheritance

*BMI* Body Mass Index, *DBP* diastolic blood pressure, *FPG* fasting plasma glucose, *HDL* high density lipoprotein cholesterol, *LDL* low density lipoprotein cholesterol, *SBP* systolic blood pressure, *TC* total cholesterol, *TG* Triglycerides, *WC* waist circumference

^a^Indicated a significant result

p-value*: p-values after Bonferroni correction

For the Bonferroni correction, p-values were multiplied by 3 (the number of SNPs in our study)

Calculations were performed using SNPassoc R package

The carriers of two copies of the rs735396 minor allele had significantly higher WC than the carriers of the reference and the heterozygous genotypes. This association remained significant after Bonferroni correction (p-value = 0.039).

The genotype distribution of the variant rs2464196 showed a significant difference between controls and MetS patients for WC (p-value = 0.035) and HDL levels (p = 0.037) after adjustment for age, sex and BMI. However, these associations were lost after Bonferroni correction. Regarding the variant rs1169288, we did not find any significant association with any trait of the MetS.

We investigated also the association of the three *HNF1A* variants with quantitative traits in the group of women and men. Our results showed that the female carriers of the rs1169288 CC minor genotype had higher cholesterol levels (mean = 5.58 mmol/l) compared to the female carriers of AA + AC genotypes (mean = 5.14 mmol/l). This association remains significant after Bonferroni correction (Additional file 5).

### Haplotype association analysis

**Fig. 1:**
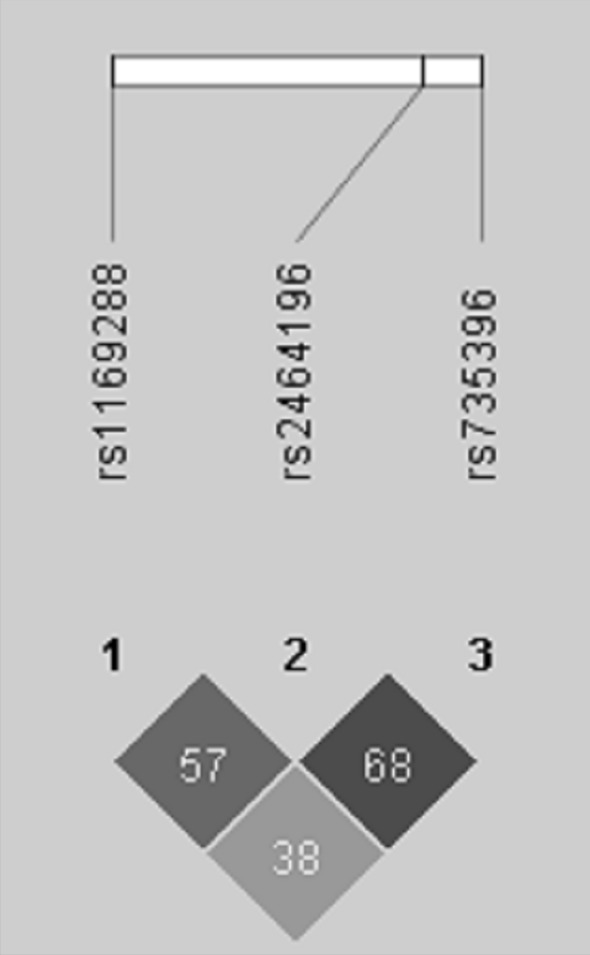
Linkage disequilibrium (LD) plot for the three genotyped *HNF1A* polymorphisms in the Tunisian study sample. Each number in the squares refers to the r^2^ coefficient of LD between the correspondent SNPs multiplied by 100. The LD plot was generated using the Haploview software (version 4.2)

The analysis of LD pattern showed low to medium correlations between the three genotyped SNPs in our study (0.38 <  = r^2^ <  = 0.68) (Fig. 1).

The haplotype analysis of the three genotyped SNPs (rs1169288, rs2464196 and rs735396) showed no significant association with MetS neither in total cohort nor in stratified groups according to the sex (Additional files 6 & 7). However, a significant association was found between the minor haplotype CAC and higher cholesterol levels in the women group (Additional file 8).

### Meta-analysis

Following our inclusion criteria, we identified only one study investigating the association of three *HNF1A* polymorphisms (rs1169288, rs2464196, rs735396) with the Mets in the Moroccan population (Table 3).

**Table 3:** Characteristics of the studies included in the meta-analysis

Study	Population	Group	Subjects	Average age (years)	rs1169288			rs2464196			rs735396		
					AA	AC	CC	GG	GA	AA	TT	TC	CC
Morjane et al. (2017)	Morocco	Controls	137	50.6 ± 10.34	82	38	10	53	57	25	39	70	26
		Cases	104	57.59 ± 11.57	39	44	14	27	44	27	39	42	23
Present study	Tunisia	Controls	299	52.56 ± 10.09	116	136	44	107	141	51	79	140	79
		Cases	295	56.4 ± 8.50	106	151	37	98	151	46	77	149	67

The study conducted by Morjane et al. in the Moroccan population reported an association of rs1169288 and rs2464196 with the risk of MetS under codominant and dominant genetic models, which was not the case in the study conducted in our Tunisian cohort. Regarding rs735396, the two studies suggested the absence of its association with MetS in both populations. Begg’s test showed that there is no publication bias for the three polymorphisms (all P > 0.05).

The meta-analysis results of both fixed and random effects models are summarized in Table 4. There was no significant association between any of the three studied *HNF1A* polymorphisms and the MetS risk.

**Table 4:** Results of meta-analysis using different genetic models

SNP	Genetic model	Fixed effects model		Random effects model		Heterogeneity	
		OR (95% CI)	p-value	OR (95% CI)	p-value	P-value	I^2^ (%)
rs1169288	AC vs AA	1.27 (0.91–1.79)	0.15	1.42 (0.68–2.95)	0.33	0.047	74.47
A > C	CC vs AA	1.14 (0.68–1.90)	0.61	1.36 (0.46–3.99)	0.57	0.058	72.16
	AC + CC vs AA	1.26 (0.91–1.73)	0.14	1.43 (0.64–3.20)	0.37	0.018	81.88
	CC vs AC + AA	1.01 (0.19–0.63)	0.94	1.12 (0.52–2.41)	0.76	0.149	51.76
rs2464196	GA vs GG	1.21 (0.85–1.71)	0.28	1.28 (0.75–2.18)	0.35	0.164	48.13
G > A	AA vs GG	1.19 (0.75–1.88)	0.44	1.39 (0.42–4.55)	0.57	0.013	83.45
	GA + AA vs GG	1.19 (0.86–1.66)	0.28	1.35 (0.65–2.82)	0.41	0.042	75.65
	AA vs GA + GG	1.07 (0.71–1.61)	0.73	1.16 (0.49–2.78)	0.72	0.039	76.52
rs735396	TC vs TT	0.84 (0.58–1.21)	0.35	0.82 (0.52–1.28)	0.39	0.234	29.18
T > C	CC vs TT	0.81 (0.53–1.24)	0.34	0.81 (0.53–1.24)	0.34	0.958	0
	TC + CC vs TT	0.83 (0.59–1.63)	0.28	0.83 (0.59–1.16)	0.28	0.389	0
	CC vs TC + TT	0.89 (0.61–1.67)	0.53	0.89 (0.61–1.27)	0.53	0.548	0

*OR* Odds ratio, *CI* Confidence interval

**Fig. 2:**
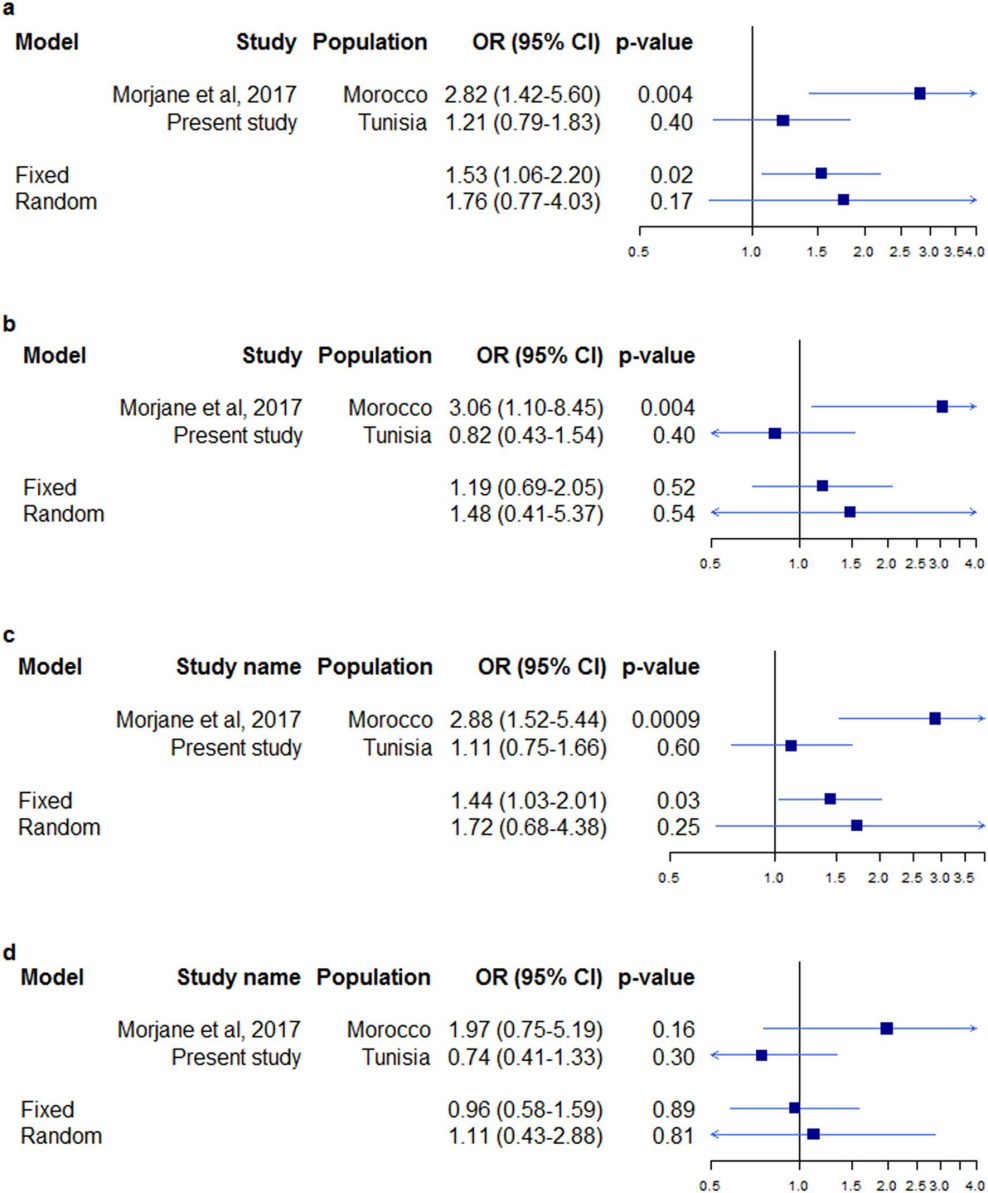
Forest plots showing the meta-analysis results of the association between rs1169288 (A > C) and MetS in women. **a** Genetic model: AC vs AA, **b** Genetic model: CC vs AA, **c** Genetic model: AC + CC vs AA, **d** Genetic model: CC vs AA + AC. The forest plots were generated using “rmeta” R library

The results of the association of the *HNF1A* polymorphisms with MetS after stratification following the sex in the Moroccan study sample are available in the Additional File 9. The meta-analysis stratified by the sex showed a significant association of rs1169288 with MetS only in the women group, under the codominant and dominant genetic models (Additional file 10 & Fig. 2). This signal was found in the fixed effects model. However, the Cochrane’s Q test p-value and I^2^ values indicate the presence of significant heterogeneity between the findings of the two studies for this polymorphism in the women group, which led us to use the result obtained in the random effects model denying the significant signal observed in the fixed effect model.

## Discussion

In the present study, we screened the association of *HNF1A* gene with the MetS, through the genotyping of three of its polymorphisms (rs1169288, rs2464196 and rs735396) in a Tunisian case/control cohort.

The activity of the HNF1A transcription factor depends on three functional domains. Variations occurring within these domains may have the biggest potential to alter its activity. The two missense genotyped variants rs1169288 and rs2464196 occur in the dimerization domain and the transactivation domain respectively. The third variant rs735396, occurring in intron 9, is localized in a transcription regulatory region [40]. The transactivation domain contains binding sites for other transcription coregulators that are important for the regulation of the target genes expression. The dimerization domain is responsible of the protein quaternary structure [41]. Hence, the combination of minor alleles of these three variants may have a major effect on HNF1A protein structure and function.

Our results showed no significant association of the genotyped SNPs with MetS in the studied sample even after stratification of the cohort following the sex and the geographic origin. The power analysis demonstrated that our study sample size (299 controls/295 cases) does not affect the detection of significant association. Indeed, we have previously identified significant associations of *APOA5* polymorphisms with the MetS in two studies performed on the same cohort of the present work [26, 27].

To our knowledge, only two studies have investigated the association of *HNF1A* gene with MetS [23, 42]. Pollex et al., reported the association of the variant G319S with MetS in the Canadian Oji-Cree isolated population [42]. However, the investigation of this variant with MetS was not replicated in other populations. The three *HNF1A* gene variants (rs1169288, rs2464196 and rs735396) were selected on the basis of several previous studies reporting the association of these SNPs with T2D and cardiovascular diseases which are related to MetS as being a component or a complication [13–20]. Our finding is different from that observed in the study performed by Morjane et al. who reported that the variants rs1169288 and rs2464196 in the *HNF1A* gene conferred an increased risk to MetS in a Moroccan population [23]. This difference may be explained by the heterogeneity of the populations’ ethnic origins.

In the second part of our work, we investigated the association of the *HNF1A* gene variants (rs1169288, rs2464196 and rs735396) with MetS quantitative traits such as FPG, BMI, LDL, HDL, SBP, DBP, TG and WC in order to assess the involvement of these variants in the MetS components. Our results showed a significant association between the variant rs735396 and higher WC after Bonferroni correction, which is in agreement with Morjane et al. study [23]. This finding suggests that rs735396 may represent a genetic susceptibility to the obesity and can lead to the alteration of some metabolic and inflammatory pathways markers such as C-reactive protein levels, as it was mentioned in previous studies [18, 19]. In this context, it is noteworthy to mention that obesity and altered CRP levels were associated with an increased risk for liver cancer [43, 44]. Furthermore, Jiang et al. had recently reported significant associations of this variant with the development of liver cancer, mainly through altering *HNF1A* gene expression in various stages of carcinogenesis. They suggested that rs735396, which is located in an enhancer regulatory region, might modulate HNF1A expression through affecting the temporal interaction between different trans-acting factors and the *HNF1A* enhancer [45]. Besides association with cancers, it was previously reported that elevation of CRP levels was associated with increased risk of ischemic stroke [46]. Therefore, effect of rs735396 on the susceptibility of ischemic stroke is worthwhile to be further investigated.

The stratified analysis, according to the sex, showed a strong association of rs1169288 genotypes with higher cholesterol levels only in the group of women. This result was not reported in the literature, and it may suggest the role of some sex-specific hormonal pathways, regulated by the HNF1A transcriptional machinery, in the metabolism of lipids. In this context, a recent study demonstrated that rs1169288 minor genotype is associated with an increased risk of preeclampsia, a pregnancy complication characterized by high blood pressure and signs of damage in liver [47]. Interestingly, Lee et al. reported an increased cholesterol biosynthesis and accumulation in the women with preeclampsia [48]. Subsequently, an in-depth investigation by Silva et al. demonstrated that inflammation intensification, resulting from an accumulation of cholesterol crystals, is the main pathway leading to preeclampsia [49]. Taking into account these points as well as the high expression of HNF1A in the liver, our findings may emphasize the potential impact of rs1169288 minor genotype in altering the cholesterol homeostasis in women. Accordingly, a previous study reported a sex-specific difference for the association of the *FTO* gene polymorphisms to MetS components in the Tunisian population [50].

Knowing that the genetic landscape of the Tunisian population is a mosaic due to successive invasions and migratory flows since the prehistoric period [51], we investigated the impact of the geographic origin on the genotype distribution of *HNF1A* variants (rs2464196, rs735396 and rs1169288). As a result, we did not find a significant association with MetS under any genotypic model neither for the Northern nor for the Southern region. In our previous study on the association of *APOA5* gene variants with MetS, we emphasized an inter-regional variation within the Tunisian population since the variant rs651821 was significantly associated with MetS only for individuals originating from Northern Tunisia [26]. This is not the case for *HNF1A* variants. The sample size does not influence the result since the two studies were performed on the same Tunisian cohort.

When investigating the haplotype association with MetS quantitative traits, we found a significant association between the minor haplotype and higher cholesterol levels mainly in the women group. In this context, Hu et al. have recently demonstrated that HNF1A modulated the cholesterol homeostasis by activating the expression of microRNA-122 (miR-122), which is an abundant liver specific microRNA that regulates hepatocyte differentiation and proliferation as well as lipid metabolism. In addition, they found that loss of HNF1A function led to an abnormal cholesterol metabolism by altering HNF1A binding to miR-122 gene promotor and downregulating its expression [52]. Similarly, Huang et al. reported low circulating miR-122 levels in diabetic patients carrying *HNF1A* variants, and they suggested that this observation might partially explain the increased risk for abnormal lipid metabolism [53]. In this context, recent studies reported that serum miR-122 is associated with insulin resistance, obesity, and MetS [54, 55]. Therefore, these findings are in agreement with our results since the genotyped SNPs rs1169288 and rs2464196 are located within functional domains of the HNF1A protein, which may affect its binding to miR-122 promotor, resulting in the alteration of the cholesterol homeostasis. In another study, Zhou et al. have demonstrated that both rs1169288 and rs2464196 were significantly associated with serum lipid levels in controls as well as in coronary artery disease patients [24]. Indeed, Willer et al. found that genetic loci associated with cholesterol levels were also associated with the risk of coronary artery disease [56]. Furthermore, a genetic variant in *HNF1A* was involved in a genetic score that is able to identify individuals at high risk of coronary heart disease, and with the largest relative and absolute clinical benefit with statin therapy, which is widely used for decreasing cholesterol levels [57]. Thus, our findings may potentially provide more insights into the study of the sex-specific biological pathways mediating lipid metabolism and involving HNF1A transcriptional machinery, which would identify potential therapeutic targets for the treatment of patients with lipid metabolism disorders. Particularly, it may be interesting to further explore the pathways affected by the statin treatment, and regulated by HNF1A. In fact, besides the effect of statin on reducing miR-122 serum levels, Li et al. have recently demonstrated that therapeutic targeting of a novel long non-coding RNA regulating HNF1A expression, might be an effective approach to enhance the effect of statin on cholesterol levels in clinics [53, 58].

Regarding the meta-analysis performed with the study carried in the Moroccan population, we did not find an association between the three *HNF1A* polymorphisms (rs1169288, rs2464196, rs735396) and the MetS. This result was expected for rs735396, since both studies have reported the same result. For the other two polymorphisms, although the study conducted in the Moroccan population reported their association with MetS in codominant and dominant models, this outcome was denied when combined with our replication study conducted in the Tunisian population. To the best of our knowledge, this is the first meta-analysis of the association of *HNF1A* polymorphisms with MetS. In fact, published meta-analyses reported especially significant signals of *HNF1A* association with T2D and diabetes related serum biomarkers [59, 60]. In North Africa, a previous meta-analysis grouping two studies from Morocco and Tunisia reported the association of one *HNF1A* variant with T2D, and ruled out the association of another variant seen only in the Moroccan study, which is similar to the outcome of our meta-analysis [61].

Our study has some possible limitations. Firstly, although the power analysis demonstrated that the sample size does not affect the detection of a significant association, it is relatively small to provide sufficient power in order to confirm the non-association between MetS and *HNF1A* genetic variants. Secondly, the stratification of the sample according to the geographic origin further decreases statistical power. Thirdly, only two studies have investigated the association of the three *HNF1A* variants (rs1169288, rs2464196, rs735396) with MetS, which did not allow to gain a great statistical power in the meta-statistical analysis.

## Conclusions

So far at the exception of two studies, no association studies have been conducted between *HNF1A* gene variants and MetS. Our results showed a significant association between the variant rs735396 and waist circumference and a significant association between the variant rs1169288 and high cholesterol level only in women. At haplotypic scale, a significant association was found between the minor haplotype and total cholesterol mainly in women. Our findings exclude the involvement of the three *HNF1A* variants rs1169288, rs2464196 and rs735396 in the susceptibility to MetS in Tunisia, but they emphasize the role of these polymorphisms in the metabolism of lipids with sex-specific differences. Nevertheless, due to the few number of studies, further case–control studies and meta-analyses are required in order to confirm the role of *HNF1A* polymorphisms in the genetic predisposition to MetS.

## Supplementary Information

**Additional file 1: Table S1**. Association of HNF1A genotypes with metabolic syndrome in the studied Tunisian population.

**Additional file 2: Table S2**. Genotypic distribution of HNF1A variants in the studied Tunisian population stratified according to the sex.

**Additional file 3: Table S3**. Genotypic distribution of HNF1A variants in the studied Tunisian population stratified following the geographic origin.

**Additional file 4: Table S4**. Genotypic distribution of HNF1A variants in the studied Tunisian population stratified following the sex and the geographic origin.

**Additional file 5: Table S5**. Association of HNF1A variants with metabolic syndrome traits for the Tunisian women in the study cohort.

**Additional file 6: Table S6**. Haplotype association analysis of the HNF1A variants with metabolic syndrome in the studied Tunisian population.

**Additional file 7: Table S7**. Haplotype analysis of the HNF1A variants with metabolic syndrome after stratification of the studied Tunisian population according to the sex.

**Additional file 8: Table S8**. Association of HNF1A minor haplotype CAC with metabolic syndrome traits in the studied Tunisian population.

**Additional file 9: Table S9**. Genotypic distribution of HNF1A variants in the Moroccan population stratified following the sex.

**Additional file 10: Table S10**. Results of meta-analysis using different genetic models in women.

## Data Availability

The datasets used and/or analysed during the current study are available from the corresponding author on reasonable request.

## Abbreviations

AIC: Akaike information criterion
BMI: Body mass index
CI: Confidence interval
DBP: Diastolic blood pressure
FPG: Fasting plamsa glucose
GWAS: Genome-wide association studies
HDL: High-density lipoprotein
HNF1A: Hepatocyte Nuclear Factor 1 Alpha
HWE: Hardy Weinberg equilibrium
LD: Linkage disequilibrium
LDL: Low-density lipoprotein
MetS: Metabolic syndrome
miR: MicroRNA
OR: Odds ratio
SBP: Systolic blood pressure
SD: Standard deviation
SNPs: Single nucleotide polymorphisms
T2D: Type 2 diabetes
TG: Triglycerides
WC: Waist circumference
