# Supplementary material for: Association of HNF1A gene variants and haplotypes with metabolic syndrome: a case–control study in the Tunisian population and a meta-analysis
Source: Diabetol Metab Syndr. 2022 Feb 2;14:25. doi: 10.1186/s13098-022-00794-0 (PMC8812021; doi:10.1186/s13098-022-00794-0)
Supplement: Supplementary file 1 — Additional file 1: Table S1. Association of HNF1A genotypes with metabolic syndrome in the studied Tunisian population. [file 13098_2022_794_MOESM1_ESM.docx]

**Supplementary Table 1** Association of *HNF1A* genotypes with metabolic syndrome in the studied Tunisian population

| Genotype distribution Codominant model Dominant model Recessive model  Control subjects (%) MetS patients (%) p-value OR (95% CI) p-value OR (95% CI) p-value OR (95% CI) p-value | | |
| --- | --- | --- |
| rs1169288 | | |
| AA  AC  CC  MAF (C)  HWE p-value  AIC | 116 (39.2%) 106 (36.1%) 0.48  136 (45.9%) 151 (51.4%) 0.21  44 (14.9%) 37 (12.6%) 0.49  0.37 0.38  0.71 0.17 | 1.02 (0.68-1.52)  0.84 (0.46-1.53) 0.8 0.98 (0.67-1.44) 0.92 0.83 (0.48-1.44) 0.5  665.3 663.9 663.5 |
| rs2464196 | | |
| GG  GA  AA  MAF (A)  HWE p-value  AIC | 107 (35.8%) 98 (33.2%) 0.56  141 (47.2%) 151 (51.2%) 0.36  51 (17.1%) 46 (15.6%) 0.71  0.406 0.411  0.71 0.4 | 1.04 (0.69-1.56)  0.79 (0.45-1.39) 0.59 0.97 (0.66-1.44) 0.89 0.77 (0.46-1.28) 0.31  670.2 669.3 668.3 |
| rs735396 | | |
| TT  TC  CC  MAF (C)  HWE p-value  AIC | 79 (26.5%) 77 (26.3%) 1.00  140 (47%) 149 (50.9) 0.39  79 (26.5%) 67 (22.9%) 0.35  0.5 0.48  0.29 0.81 | 0.98 (0.63-1.53)  0.82 (0.49-1.38) 0.68 0.92 (0.61-1.40) 0.71 0.83 (0.54-1.28) 0.39  667.6 666.2 665.6 |

MetS: metabolic syndrome patients; Genotype distributions are shown as number (%); OR: Odds Ratio, 95% CI: 95% Confidence intervals; HWE: Hardy-Weinberg equilibrium; MAF: Minor Allele Frequency; AIC: Akaike Information Criterion; p-values are adjusted for age, sex and BMI. Calculations were performed using SNPassoc R package.
