# Supplementary material for: Association of HNF1A gene variants and haplotypes with metabolic syndrome: a case–control study in the Tunisian population and a meta-analysis
Source: Diabetol Metab Syndr. 2022 Feb 2;14:25. doi: 10.1186/s13098-022-00794-0 (PMC8812021; doi:10.1186/s13098-022-00794-0)
Supplement: Supplementary file 2 — Additional file 2: Table S2. Genotypic distribution of HNF1A variants in the studied Tunisian population stratified according to the sex. [file 13098_2022_794_MOESM2_ESM.docx]

**Supplementary Table 2** Genotypic distribution of *HNF1A* variants in the studied Tunisian population stratified according to the sex

| Genotype distribution Codominant model Dominant model Recessive model  Control subjects (%) Mets patients (%) OR (95% CI) p-value OR (95% CI) p-value OR (95% CI) p-value | | |
| --- | --- | --- |
| Women | | |
| rs1169288  AA  AC  CC | 88 (40.6%) 73 (35.8%)  98 (45.2%) 98 (51.2%)  31 (14.3%) 21 (10.9%) | 1.21 (0.79-1.83)  0.82 (0.43-1.54) 0.40 1.11 (0.75-1.66) 0.60 0.74 (0.41-1.33) 0.30 |
| rs2464196  GG  GA  AA | 78 (35.8%) 65 (33.7%)  102 (46.8%) 101 (52.3%)  38 (17.4%) 27 (14.0%) | 1.19 (0.77-1.82)  0.85 (0.47-1.54) 0.46 1.10 (0.73-1.65) 0.65 0.77 (0.45-1.32) 0.33 |
| rs735396  TT  TC  CC | 58 (26.7%) 52 (27.1%)  102 (47%) 96 (50%)  57 (26.3%) 44 (22.9%) | 1.05 (0.66-1.67)  0.86 (0.50-1.48) 0.72 0.98 (0.63-1.52) 0.93 0.83 (0.53-1.31) 0.43 |
| Men | | |
| rs1169288  AA  AC  CC | 28 (35.4%) 31 (32.6%)  38 (48.1%) 49 (51.6%)  13 (16.5%) 15 (15.8%) | 1.16 (0.60-2.26)  1.04 (0.42-2.57) 0.89 1.13 (0.60-2.13) 0.69 0.95 (0.42-2.14) 0.90 |
| rs2464196  GG  GA  AA | 29 (35.8%) 32 (33.7%)  39 (48.1%) 44 (46.3%)  13 (16%) 19 (20%) | 1.02 (0.53-1.98)  1.32 (0.56-3.15) 0.79 1.10 (0.59-2.05) 0.76 1.31 (0.60-2.85) 0.49 |
| rs735396  TT  TC  CC | 21 (25.9%) 25 (26.6%)  38 (46.9%) 46 (48.9%)  22 (27.2%) 23 (24.5%) | 1.02 (0.49-2.09)  0.88 (0.39-2.00) 0.92 0.97 (0.49-1.90) 0.92 0.87 (0.44-1.71) 0.68 |

MetS: metabolic syndrome patients; Genotype distributions are shown as number (%), OR: Odds Ratio, 95% CI: 95% Confidence intervals

p-values are generated by simple logistic regression carried out using SNPassoc R package.
