# Supplementary material for: Association of HNF1A gene variants and haplotypes with metabolic syndrome: a case–control study in the Tunisian population and a meta-analysis
Source: Diabetol Metab Syndr. 2022 Feb 2;14:25. doi: 10.1186/s13098-022-00794-0 (PMC8812021; doi:10.1186/s13098-022-00794-0)
Supplement: Supplementary file 3 — Additional file 3: Table S3. Genotypic distribution of HNF1A variants in the studied Tunisian population stratified following the geographic origin. [file 13098_2022_794_MOESM3_ESM.docx]

**Supplementary Table 3** Genotypic distribution of *HNF1A* variants in the studied Tunisian population stratified following the geographic origin

| Genotype distribution Codominant model Dominant model Recessive model  Control subjects (%) Mets patients (%) OR (95% CI) p-value OR (95% CI) p-value OR (95% CI) p-value | | |
| --- | --- | --- |
| Northern population | | |
| rs1169288  AA  AC  CC | 82 (37.8%) 86 (35.8%)  106 (48.8%) 127 (52.9%)  29 (13.4%) 27 (11.2%) | 1.02 (0.68-1.52)  0.68 (0.35-1.33) 0.45 0.95 (0.63-1.44) 0.8 0.67 (0.36-1.25) 0.21 |
| rs2464196  GG  GA  AA | 75 (34.2%) 79 (32.8%)  109 (49.8%) 124 (51.5%)  35 (16%) 38 (15.8%) | 1.05 (0.67-1.64)  0.69 (0.37-1.29) 0.36 0.96 (0.63-1.46) 0.83 0.67 (0.39-1.17) 0.16 |
| rs735396  TT  TC  CC | 55 (25.1%) 62 (25.9%)  110 (50.2%) 121 (50.6%)  54 (24.7%) 56 (23.4%) | 0.91 (0.56-1.49)  0.80 (0.45-1.42) 0.73 0.88 (0.55-1.39) 0.57 0.85 (0.53-1.36) 0.49 |
| Southern population | | |
| rs1169288  AA  AC  CC | 7 (36.8%) 9 (37.5%)  11 (57.9%) 11 (45.8%)  1 (5.3%) 4 (16.7%) | 1.07 (0.26-4.51)  7.96 (0.28-228.12) 0.36 1.35 (0.34-5.41) 7.62 (0.30-193.38) 0.13 |
| rs2464196  GG  GA  AA | 7 (38.9%) 9 (37.5%)  11 (61.1%) 11 (45.8%)  1 (0%) 4 (16.7%) | 1.04 (0.69-1.56)  0.79 (0.45-1.39) 0.59 0.97 (0.66-1.44) 0.89 0.77 (0.46-1.28) 0.31 |
| rs735396  TT  TC  CC | 6 (31.6%) 8 (33.3%)  8 (42.1%) 10 (41.7%)  5 (26.3%) 6 (25%) | 1.38 (0.29-6.60)  1.27 (0.19-8.43) 0.92 1.34 (0.32-5.67) 0.68 1.07 (0.20-5.76) 0.93 |

MetS: metabolic syndrome patients; Genotype distributions are shown as number (%), OR: Odds Ratio, 95% CI: 95% Confidence intervals; p-values are generated by multivariate logistic regression adjusted for age, sex and BMI. Significant p-values (<0.05). Calculations were performed using SNPassoc R library.
