# Supplementary material for: Association of HNF1A gene variants and haplotypes with metabolic syndrome: a case–control study in the Tunisian population and a meta-analysis
Source: Diabetol Metab Syndr. 2022 Feb 2;14:25. doi: 10.1186/s13098-022-00794-0 (PMC8812021; doi:10.1186/s13098-022-00794-0)
Supplement: Supplementary file 4 — Additional file 4: Table S4. Genotypic distribution of HNF1A variants in the studied Tunisian population stratified following the sex and the geographic origin. [file 13098_2022_794_MOESM4_ESM.docx]

**Supplementary Table 4** Genotypic distribution of *HNF1A* variants in the studied Tunisian population stratified following the sex and the geographic origin

| Genotype distribution Codominant model Dominant model Recessive model  Women (%) Men (%) OR (95% CI) p-value OR (95% CI) p-value OR (95% CI) p-value | | |
| --- | --- | --- |
| Northern population | | |
| rs1169288  AA  AC  CC | 123 (38.6%) 46 (35.8%)  160 (50.2%) 74 (52.9%)  36 (11.3%) 22 (11.2%) | 1.24 (0.80-1.91)  1.63 (0.87-3.07) 0.29 1.31 (0.86-1.99) 0.20 1.44 (0.81-2.55) 0.21 |
| rs2464196  GG  GA  AA | 109 (34.1%) 46 (31.9%)  164 (51.2%) 70 (48.6%)  47 (14.7%) 28 (19.4%) | 1.01 (0.65-1.58)  1.41 (0.79-2.52) 0.44 1.10 (0.72-1.67) 0.65 1.40 (0.84-2.35) 0.20 |
| rs735396  TT  TC  CC | 85 (26.6%) 33 (23.1%)  159 (49.8%) 73 (51%)  75 (23.5%) 37 (25.9%) | 1.18 (0.73-1.93)  1.27 (0.72-2.23) 0.68 1.21 (0.76-1.92) 0.41 1.14 (0.72-1.79) 0.58 |
| Southern population | | |
| rs1169288  AA  AC  CC | 27 (47.4%) 7 (35%)  21 (36.8%) 10 (50%)  9 (15.8%) 3 (15%) | 1.84 (0.60-5.64)  1.29 (0.27-6.05) 0.56 1.67 (0.58-4.80) 0.33 0.94 (0.23-3.89) 0.93 |
| rs2464196  GG  GA  AA | 24 (41.4%) 9 (45%)  22 (37.9%) 9 (45%)  12 (20.7%) 2 (10%) | 1.09 (0.37-3.25)  0.44 (0.08-2.39) 0.52 0.86 (0.31-2.40) 0.77 0.43 (0.09-2.09) 0.25 |
| rs735396  TT  TC  CC | 19 (33.3%) 7 (35%)  21 (36.8%) 8 (40%)  17 (29.8%) 5 (25%) | 1.03 (0.31-3.40)  0.80 (0.21-2.99) 0.91 0.93 (0.32-2.71) 0.89 0.78 (0.25-2.50) 0.67 |

Genotype distributions are shown as number (%), OR: Odds Ratio, 95% CI: 95% Confidence intervals; p-values are generated by simple logistic regression. Significant p-values (<0.05). Calculations were performed using SNPassoc R library.
