# Supplementary material for: Association of HNF1A gene variants and haplotypes with metabolic syndrome: a case–control study in the Tunisian population and a meta-analysis
Source: Diabetol Metab Syndr. 2022 Feb 2;14:25. doi: 10.1186/s13098-022-00794-0 (PMC8812021; doi:10.1186/s13098-022-00794-0)
Supplement: Supplementary file 5 — Additional file 5: Table S5. Association of HNF1A variants with metabolic syndrome traits for the Tunisian women in the study cohort. [file 13098_2022_794_MOESM5_ESM.docx]

**Supplementary Table 5** Association of *HNF1A* variants with metabolic syndrome traits for the Tunisian women in the study cohort

|  | rs1169288 | rs2464196 | rs735396 |
| --- | --- | --- | --- |
|  | AA+AC CC p-value p-value* | GG+GA AA p-value p-value* | TT+TC CC p-value p-value* |
| WC (cm)  BMI (kg/m^2^)  FPG (mmol/l)  SBP (mmHg)  DBP (mmHg)  TC (mmol/l)  HDL (mmol/l)  LDL (mmol/l)  TG (mmol/l) | 101.7 102.4 0.74 1  30.88 31.57 0.41 1  7.69 8.18 0.41 1  13.91 13.73 0.60 1  8.02 8.02 0.98 1  5.14 5.58 0.005^a^ 0.015^a^  1.36 1.46 0.13 0.39  3.26 3.47 0.20 0.60  1.57 1.64 0.58 1 | 101.2 104.9 0.035^a^ 0.105  30.7 32.4 0.027^a^ 0.081  7.68 8.12 0.41 1  13.86 14.06 0.54 1  8.01 8.11 0.60 1  5.14 5.45 0.035^a^ 0.105  1.35 1.48 0.036^a^ 0.108  3.27 3.39 0.39 1  1.57 1.64 0.58 1 | 101 104.3 0.033^a^ 0.099  30.78 31.52 0.24 0.72  7.79 7.60 0.68 1  13.88 13.93 0.86 1  8.05 7.94 0.50 1  5.18 5.21 0.78 1  1.35 1.45 0.07 0.21  3.27 3.33 0.61 1  1.58 1.68 0.88 1 |

Data are presented as means. Linear regression was used to assess genotype/phenotype correlations under the recessive model of inheritance for the women.

WC: waist circumference; BMI: Body mass index; FPG: Fasting plasma glucose; SBP: Systolic blood pressure; DBP: Diastolic blood pressure; TC: Total cholesterol; HDL: High density lipoprotein; LDL: Low density lipoprotein; TG: Triglycerides.

^a^ indicated a significant result.

p-value*: p-values after Bonferroni correction.

Calculations were performed using SNPassoc R package.
