# Supplementary material for: Association of HNF1A gene variants and haplotypes with metabolic syndrome: a case–control study in the Tunisian population and a meta-analysis
Source: Diabetol Metab Syndr. 2022 Feb 2;14:25. doi: 10.1186/s13098-022-00794-0 (PMC8812021; doi:10.1186/s13098-022-00794-0)
Supplement: Supplementary file 6 — Additional file 6: Table S6. Haplotype association analysis of the HNF1A variants with metabolic syndrome in the studied Tunisian population. [file 13098_2022_794_MOESM6_ESM.docx]

**Supplementary Table 6** Haplotype association analysis of the genotyped *HNF1A* variants with metabolic syndrome in the studied Tunisian population

| Haplotype | Frequency in total cohort | Frequency in cases | Frequency in controls | OR | p-value |
| --- | --- | --- | --- | --- | --- |
| CAC | 0.337 | 0.3463 | 0.3303 | 1.08 | 0.523 |
| AAC | 0.0679 | 0.0646 | 0.07206 | 0.893 | 0.625 |
| AGC | 0.0861 | 0.0721 | 0.09985 | 0.7 | 0.0932 |
| CGT | 0.0409 | 0.03715 | 0.04497 | 0.811 | 0.494 |
| AGT | 0.464 | 0.4799 | 0.4528 | 1.13 | 0.303 |

Haplotype order: rs1169288 (A>C), rs2464196 (G>A**)**, rs735396 (T>C)

OR: Odds Ratio.

Haplotype association analysis was performed using plink software (version 1.07).
