# Supplementary material for: Association of HNF1A gene variants and haplotypes with metabolic syndrome: a case–control study in the Tunisian population and a meta-analysis
Source: Diabetol Metab Syndr. 2022 Feb 2;14:25. doi: 10.1186/s13098-022-00794-0 (PMC8812021; doi:10.1186/s13098-022-00794-0)
Supplement: Supplementary file 7 — Additional file 7: Table S7. Haplotype analysis of the HNF1A variants with metabolic syndrome after stratification of the studied Tunisian population according to the sex. [file 13098_2022_794_MOESM7_ESM.docx]

**Supplementary Table 7** Haplotype association analysis of the genotyped *HNF1A* variants with metabolic syndrome after stratification of the studied Tunisian population according to the sex

| Haplotype | Frequency in total cohort | Frequency in cases | Frequency in controls | OR | p-value |
| --- | --- | --- | --- | --- | --- |
| Women | | | | | |
| CAC | 0.326 | 0.332 | 0.326 | 1.04 | 0.813 |
| AAC | 0.072 | 0.068 | 0.076 | 0.89 | 0.666 |
| AGC | 0.086 | 0.077 | 0.095 | 0.78 | 0.352 |
| CGT | 0.033 | 0.030 | 0.037 | 0.79 | 0.595 |
| AGT | 0.471 | 0.490 | 0.463 | 1.13 | 0.394 |
| Men | | | | | |
| CAC | 0.354 | 0.364 | 0.341 | 1.1 | 0.658 |
| AAC | 0.063 | 0.067 | 0.059 | 1.14 | 0.775 |
| AGC | 0.077 | 0.054 | 0.104 | 0.53 | 0.106 |
| CGT | 0.055 | 0.051 | 0.060 | 0.84 | 0.713 |
| AGT | 0.45 | 0.463 | 0.433 | 1.13 | 0.575 |

Haplotype order: rs1169288 (A>C), rs2464196 (G>A**)**, rs735396 (T>C), OR: Odds Ratio.

Haplotype association analysis was realized using plink software (version 1.07).
