# Supplementary material for: Association of HNF1A gene variants and haplotypes with metabolic syndrome: a case–control study in the Tunisian population and a meta-analysis
Source: Diabetol Metab Syndr. 2022 Feb 2;14:25. doi: 10.1186/s13098-022-00794-0 (PMC8812021; doi:10.1186/s13098-022-00794-0)
Supplement: Supplementary file 8 — Additional file 8: Table S8. Association of HNF1A minor haplotype CAC with metabolic syndrome traits in the studied Tunisian population. [file 13098_2022_794_MOESM8_ESM.docx]

**Supplementary Table 8** Association of *HNF1A* minor haplotype CAC with metabolic syndrome traits in the studied Tunisian population

|  | Total cohort | Women | Men |
| --- | --- | --- | --- |
| WC (cm)  BMI (kg/m^2^)  FPG (mmol/l)  SBP (mmHg)  DBP (mmHg)  TC (mmol/l)  HDL (mmol/l)  LDL (mmol/l)  TG (mmol/l) | 0.13  0.43  0.60  0.57  0.70  0.028^a^  0.77  0.32  0.80 | 0.19  0.17  0.98  0.57  0.48  0.007^a^  0.31  0.10  0.30 | 0.45  0.86  0.44  0.95  0.70  0.98  0.51  0.56  0.20 |

Haplotype order: rs1169288 (A>C), rs2464196 (G>A**)**, rs735396 (T>C).

WC: waist circumference; BMI: Body mass index; FPG: Fasting plasma glucose; SBP: Systolic blood pressure; DBP: Diastolic blood pressure; TC: Total cholesterol; HDL: High-density lipoprotein; LDL: Low-density lipoprotein; TG: Triglycerides.

^a^ indicates a significant result.

Values in the table refer to p-values of the associations of the minor haplotype CAC with metabolic syndromic traits. These p-values were computed using a generalized linear model from the haplo.score function incorporated in the haplo.stats R package.
