# Supplementary material for: Association of HNF1A gene variants and haplotypes with metabolic syndrome: a case–control study in the Tunisian population and a meta-analysis
Source: Diabetol Metab Syndr. 2022 Feb 2;14:25. doi: 10.1186/s13098-022-00794-0 (PMC8812021; doi:10.1186/s13098-022-00794-0)
Supplement: Supplementary file 9 — Additional file 9: Table S9. Genotypic distribution of HNF1A variants in the Moroccan population stratified following the sex. [file 13098_2022_794_MOESM9_ESM.docx]

**Supplementary Table 9** Genotypic distribution of *HNF1A* variants in the Moroccan population stratified following the sex

| Genotype distribution Codominant model Dominant model Recessive model  Control subjects (%) Mets patients (%) OR (95% CI) p-value OR (95% CI) p-value OR (95% CI) p-value | | |
| --- | --- | --- |
| Women | | |
| rs1169288  AA  AC  CC | 60 (63.8%) 27 (38%)  26 (27.7%) 33 (46.5%)  8 (8.5%) 11 (15.5%) | 2.82 (1.42-5.60)  3.06 (1.10-8.45) 0.004^a^ 2.88 (1.52-5.44) 0.0009^a^ 1.97 (0.75-5.19) 0.16 |
| rs2464196  GG  GA  AA | 34 (34.3%) 18 (24.7%)  47 (47.5%) 35 (47.9%)  18 (18.2%) 20 (27.4%) | 1.41 (0.68-2.89)  2.10 (0.89-4.94) 0.23 1.60 (0.81-3.14) 0.16 1.70 (0.82-3.51) 0.15 |
| rs735396  TT  TC  CC | 25 (25.3%) 26 (34.2%)  56 (56.6%) 34 (44.7%)  18 (18.2%) 16 (21.1%) | 0.58 (0.29-1.17)  0.85 (0.36-2.04) 0.28 0.65 (0.34-1.25) 0.19 1.20 (0.57-2.54) 0.63 |
| Men | | |
| rs1169288  AA  AC  CC | 23 (62.2%) 12 (46.2%)  12 (32.4%) 11 (42.3%)  2 (5.4%) 3 (11.5%) | 1.76 (0.60-5.15)  2.87 (0.42-19.62) 0.40 1.92 (0.69-5.30) 0.20 2.28 (0.35-14.74) 0.37 |
| rs2464196  GG  GA  AA | 19 (51.4%) 9 (36%)  11 (29.7%) 9 (36%)  7 (18.7%) 7 (28%) | 1.73 (0.53-5.65)  2.11 (0.57-7.86) 0.46 1.88 (0.66-5.31) 0.23 1.67 (0.50-5.53) 0.40 |
| rs735396  TT  TC  CC | 14 (37.8%) 13 (46.4%)  15 (40.5%) 8 (28.6%)  8 (21.6%) 7 (25%) | 0.57 (0.18-1.80)  0.94 (0.27-3.34) 0.60 0.70 (0.26-1.90) 0.48 1.21 (0.38-3.85) 0.74 |

OR: Odds Ratio, 95% CI: 95% Confidence intervals.

p-values are generated by logistic regression carried out using SNPassoc R package.

^a^ indicates a significant result.
