# Supplementary material for: Association of HNF1A gene variants and haplotypes with metabolic syndrome: a case–control study in the Tunisian population and a meta-analysis
Source: Diabetol Metab Syndr. 2022 Feb 2;14:25. doi: 10.1186/s13098-022-00794-0 (PMC8812021; doi:10.1186/s13098-022-00794-0)
Supplement: Supplementary file 10 — Additional file 10: Table S10. Results of meta-analysis using different genetic models in women. [file 13098_2022_794_MOESM10_ESM.docx]

**Supplementary Table 10** Results of meta-analysis using different genetic models in women

| SNP | Genetic model | Fixed effects model  OR (95% CI) p-value | Random effects model  OR (95% CI) p-value | Heterogeneity  p-value I^2^ (%) |
| --- | --- | --- | --- | --- |
| rs1169288  A>C | AC vs AA  CC vs AA  AC+CC vs AA  CC vs AC+AA | 1.53 (1.06-2.20) 0.02^a^  1.19 (0.69-2.05) 0.52  1.44 (1.03-2.01) 0.03^a^  0.96 (0.58-1.59) 0.89 | 1.76 (0.77-4.03) 0.17  1.48 (0.41-5.37) 0.54  1.72 (0.68-4.38) 0.25  1.11 (0.43-2.88) 0.81 | 0.040 76.27  0.032 78.03  0.012 83.90  0.089 65.21 |
| rs2464196  G>A | GA vs GG  AA vs GG  GA+AA vs GG  AA vs GA+GG | 1.24 (0.85-1.80) 0.25  1.13 (0.69-1.85) 0.60  1.21 (0.85-1.72) 0.27  1.01 (0.66-1.56) 0.93 | 1.24 (0.85-1.80) 0.25  1.26 (0.52-3.04) 0.60  1.21 (0.85-1.72) 0.27  1.09 (0.50-2.37) 0.81 | 0.69 0  0.089 65.37  0.355 0  0.086 65.96 |
| rs735396  T>C | TC vs TT  CC vs TT  TC+CC vs TT  CC vs TC+TT | 0.87 (0.59-1.28) 0.49  0.84 (0.54-1.35) 0.51  0.86 (0.59-1.23) 0.41  0.91 (0.62-1.34) 0.65 | 0.82 (0.46-1.46) 0.51  0.85 (0.54-1.35) 0.51  0.85 (0.58-1.24) 0.42  0.91 (0.62-1.34) 0.65 | 0.163 48.56  0.981 0  0.304 5.01  0.405 0 |

^a^ indicates a significant result.

Meta-analysis was performed using GWAMA software (version 2.2.2).
